# Clinical characteristics, pathology features and outcomes of pediatric myeloid sarcoma: A retrospective case series

**DOI:** 10.3389/fped.2022.927894

**Published:** 2022-12-05

**Authors:** Fanghua Ye, Hui Zhang, Wen Zhang, Jiajia Dong, Wenjun Deng, Liangchun Yang

**Affiliations:** Department of Pediatrics, Xiangya Hospital Central South University, Changsha, China

**Keywords:** pediatric, myeloid sarcoma, clinical characteristics, pathology, acute myeloid leukemia

## Abstract

**Purpose:**

Myeloid sarcoma (MS) is a rare extramedullary mass with myeloid expression, which is easy to be missed and misdiagnosed, especially in the pediatric population. We analyze the clinicopathological characteristics, immunophenotypic, cytogenetic, and molecular studies, therapeutic approaches, and outcomes, to optimize the management of such patients.

**Methods:**

A retrospective, single-center, case series study of eleven children diagnosed with MS by pathology was performed.

**Results:**

The male-to-female ratio was 8:3, and the median age at diagnosis was 7 years. The most commonly involved sites were the skin and orbital region, followed by lymph nodes, central nervous system, and testis. Seven cases (64%) with *Class I*-MS and four cases (36%) presented as *Class II*-MS. Immunohistochemically, MPO and CD117 were the most commonly expressed markers, followed by CD33, CD43, CD34, CD68, and lysozyme. Chromosomal abnormalities were detected in 4 patients. Two patients had the presence of deleterious mutations (FLT3, ASXL, KIT, and DHX15) on molecular detection. Ten patients were treated with chemotherapy based on AML regimens. The median follow-up time was 33.5 months in eleven patients. Two patients relapsed, one died, and one lost to follow-up. The 2-year overall survival (OS) rate estimated by Kaplan-Meier curves was 90.9% ± 8.7%, and the event-free survival (EFS) rate was 64.9% ± 16.7%.

**Conclusions:**

MS diagnosis is usually challenging. Adequate tumor biopsy and expanded immunohistochemistry are necessary for the correct diagnosis of MS. Early and regular systemic chemotherapy promises long-term survival.

## Introduction

Myeloid sarcoma (MS) is a distinct subtype of hematologic malignancy, defined by the WHO in 2016 as a mass that appears in an anatomical location outside the bone marrow containing myeloid blast cells, commonly involving the skin, soft tissues, central nervous system (CNS), and the urogenital tract (1). MS first described chloroma because of the greenish color, caused by the presence of myeloperoxidase (MPO). The terms granulocytic sarcoma, extramedullary myeloid cell tumor, or myeloblastoma have also been used (2, 3). There are no definitive data on the incidence of MS in the general population. The incidence of MS in acute myeloid leukemia (AML) patients is 2.5%–9.1%, and some literature has also reported that the rate is as high as 40% in children (4, 5). MS may present *de novo*, may accompany peripheral blood and marrow involvement may present as a relapse of AML, or may present as the progression of a prior myelodysplastic syndrome (MDS), myeloproliferative neoplasms (MPN), or MDS/MPN (1, 6).

The diagnosis of MS is based on clinical manifestations, imaging, mass biopsy, immunohistochemistry, cytogenetics, and molecular biology (7). There are few study reports on MS and no studies on large series. In most patients with known myeloproliferative disorders, the diagnosis of MS may be relatively simple. However, the diagnosis of isolated MS is a challenge.

Here, we report a comprehensive study of MS, including their clinical and laboratory manifestations, pathological and genetic features, treatments, and outcomes, in a series of 11 children. Literature review and single-center experience are used to improve pediatricians' understanding of the disease.

## Materials and methods

### Research subjects

This study was approved by the ethics committee of Xiangya Hospital Central South University in accordance with the Helsinki Declaration. Eleven children diagnosed with MS by pathology of the tumor mass were admitted from January 1, 2016, to April 1, 2022. Diagnoses were reconfirmed by two pathologists. The data we collected included the age, gender, clinical manifestations, site, history of AML or other neoplastic disorders, laboratory inspection, pathologic characteristics, radiological findings, treatment, and outcome.

### Type of diagnosis

WHO classifies them according to their extramedullary manifestations (1): *Class I*, AML with MS; *Class II*, isolated MS (normal peripheral blood smear and bone marrow morphology and no history of myeloid neoplasm); *Class III*, extramedullary relapse of AML, including relapse after bone marrow transplantation; *Class IV*, acute/transformed stage of MDS/MPNs.

### Histology, immunohistochemistry (IHC), and flow cytometry

The initial diagnosis was made on core or surgical biopsy specimens. Biopsy samples were consistent with the diagnostic criteria of MS (*A*. Morphological features: The morphology of MS manifests differently depending on the degree of myeloid differentiation, typically showing different stages of myeloid cell infiltration. *B*. Immunohistochemistry: it mostly showed MPO, CD43, CD34, CD13, CD33, CD117, CD14, CD11b, CD11c, CD68, CD163, lysozyme, and other myeloid and monocyte antigens positive, generally not expressing lymphocyte markers such as CD3, CD19, CD20) (8). Patients with bone marrow and cerebrospinal fluid (CSF) involvement were analyzed by flow cytometry included markers for CD20, CD117, CD34, CD33, CD10, CD45, HLA-DR, CD19, CD15, CD13, CD64, CD7, CD14, CD3, CD123, CD4, CD5, CD11b, and CD38.

### Chromosome karyotype and molecular testing

The G-banding technique was used to analyze the karyotype. Fluorescent *in situ* hybridization (FISH) panel including probes for deletion (5)/-5, deletion (7)/-7, t (8;21), t (15;17), and inversion (16), etc. histone lysine [K]-MethylTransferase 2A (*KMT2A*) gene formerly known as mixed-lineage leukemia (*MLL*) gene. Polymerase chain reaction (PCR) was used to detect fusion genes including *MLL/AF10, AML1/ETO, PML/RARα, ET6V/RUNX1, BCR/ABL1*, etc. Myeloid malignancies mutation panel detected in MS tumor mass or marrow by next-generation sequencing (NGS), such as fms like tyrosine kinase 3 (*FLT3*), tyrosine kinase receptor protein family *KIT* gene, nucleophosmin 1 (*NPM1*), CCAAT/enhancer binding protein alpha (*CEBPA*), additional sex combs-like 1 (*ASXL1*), etc.

### Follow-up

Patients with MS were followed up through inpatient records, outpatient visits, and telephone calls, with a deadline of April 1, 2022. Overall survival (OS) was calculated as the time from diagnosis of MS until death or last follow-up. Event-free survival (EFS) was defined as the duration between diagnosis to the occurrence of events such as relapse, progression, death for any reason, or last follow-up.

### Statistical analysis

Descriptive statistics were performed for clinicopathological, molecular characteristics, and radiologic attributes. The quantitative data were represented by median (numerical range), while the qualitative data were represented by a number of cases (%). OS and EFS were calculated with the Kaplan–Meier method.

## Results

### Clinical characteristics

The major clinical features of all cases were summarized in Table 1. Eight males and three females with a median age were 7 years (range 1–13 years). All patients had painless masses or occupying lesions located in the skin, orbital region, lymph nodes central nervous system, testis, or mediastinum. Orbital masses showed symptoms of proptosis or vision loss due to compression. Mediastinal masses caused coughs and polypnea. Only two patients (18%) had their first visit to a hematology department while nine (82%) were non-hematology departments.

**Table 1:** Clinical characteristics and outcomes of the patients with MS.

No.	Age at diagnosis	Gender	Site	First Visit Department	Previous history	Diagnosis type	Treatment	Relapse	Outcomes, months (OS/EFS)
1	3 year 6 month	Female	Temporal lobe	Ophthalmology	No	*Class II*	Surgery + Chemotherapy	No	Alive (3.5+/3.5+)
2	7 year	Male	Orbit	Ophthalmology	No	*Class I*	Surgery + Chemotherapy + HSCT + TKI	No	Alive (22+/22+)
3	7 year	Male	Orbit	Ophthalmology	No	*Class I*	Surgery + Chemotherapy	Yes (Orbit and bone marrow)	Alive (50.5+/11)
4	2 year 5 month	Female	Skin (chest), Mediastinum	Surgery	No	*Class II*	Surgery + Chemotherapy	No	Alive (36+/36+)
5	6 year 4 month	Female	Skin (lower limb soft tissue)	Surgery	No	*Class I*	Surgery + Chemotherapy	No	Alive (33.5+/33.5+)
6	1 year 10 month	Male	Forehead lobe and Temple lobe	Surgery	No	*Class I*	Surgery + Chemotherapy	No	Alive (56+/56+)
7	1 year	Male	Orbit	Ophthalmology	No	*Class I*	Surgery	–	Died (1/1)
8	12 year	Male	Cervical lymph node	Hematology	No	*Class I*	Chemotherapy + HSCT	Yes (bone marrow)	Alive (53+/15)
9	9 year	Male	Skin (maxillofacial region), Testis, Mediastinum	Stomatology	No	*Class I*	Surgery + Chemotherapy + HSCT	No	Alive (75+/75+)
10	13 year	Male	Cervical lymph node	Hematology	No	*Class II*	Chemotherapy	–	Lost (4+/4+)
11	9 year	Male	Testis, CSF	Urology surgery	No	*Class II*	Surgery + Chemotherapy + TKI	No	Alive (4+/4+)

MS, myeloid sarcoma; AML, acute myeloid leukemia; HSCT, hematopoietic stem cell transplantation; TKI, Tyrosine kinase inhibitors; CSF, cerebrospinal fluid. OS, overall survival; EFS, event-free survival; + is censored data, indicating that the patient is still alive/event-free or lost to follow-up.

### Radiological findings

**Figure 1:**
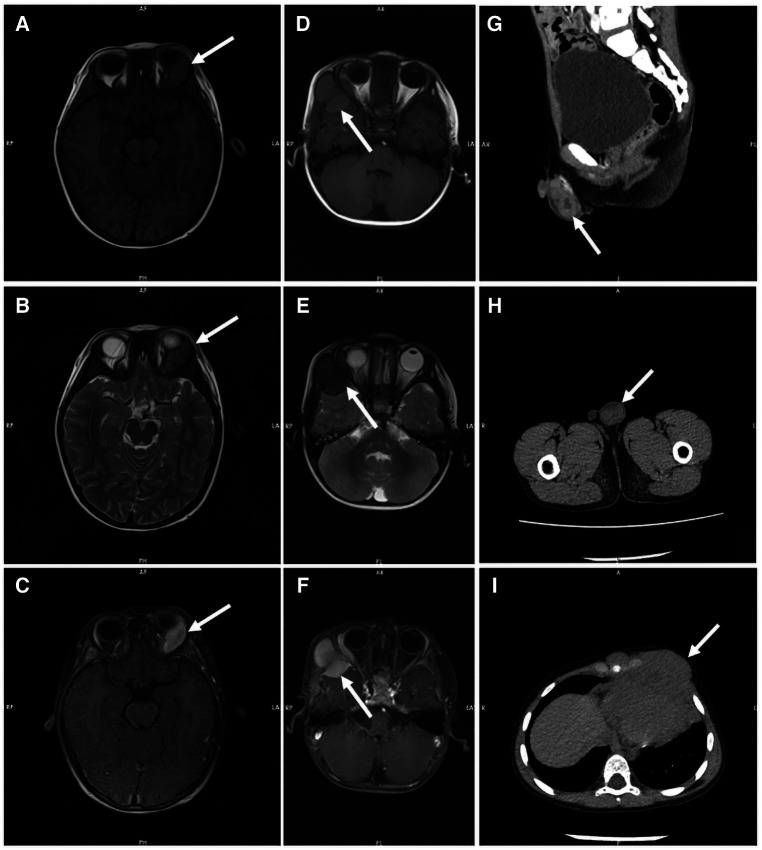
Radiological data on part patients. Both T1-weighted MR images (**A**) and T2-weighted MR images (**B**) showed equal signal intensity in the left orbital region of patient 2, which was significantly enhanced by contrast agent injection (**C**). T1 weighted MR images showed equal signal intensity (**D**), and T2 weighted MR images showed low signal intensity (**E**) in the frontal and temporal lobes of patient 6, with uniform enhancement and adjacent brain parenchymal compression (**F**). CT showed mass soft tissue density foci from the testis of patient 11 (**G and H**) and the mediastinum of patient 9 (**I**).

All patients were evaluated by Computed Tomography (CT) or Magnetic Resonance Imaging (MRI) to identify the lesion site. Multi-site involvement in four patients (36%) and single-site in seven (64%). CT showed soft tissue density foci with uneven signal and irregular boundary in most patients. Bone necrosis was seen in some patients. MRI had no specific manifestations. T1-weighted images were mostly showed an equal or low signal, while T2-weighted mainly low signal, enhanced after contrast injection. Radiological data on part patients is shown in Figure 1.

### Laboratory findings

**Figure 2:**
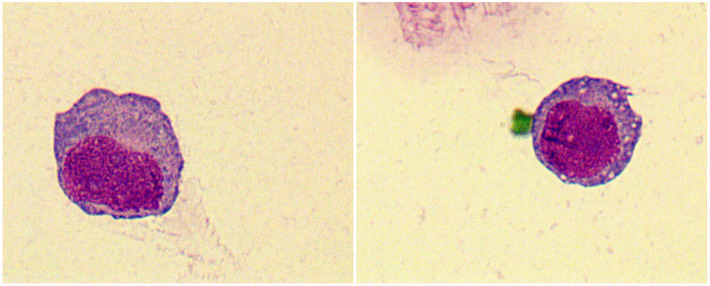
Tumor cells were found in the cerebrospinal fluid (CSF) of patient 11, which have large cytosol and a high amount of envelope. Vacuoles, fine granules and pseudopods were observed in partial cells. The nucleus was large and visibly folded and distorted. Original magnification: 1000×.

Laboratory data is found in Table 2. Three patients (27%) had anemia or thrombocytopenia at MS diagnosis. Four patients (36%) had leukocytosis and positive blasts in peripheral blood. One patient with positive CSF blasts (Figure 2). Seven patients (64%) were *Class I*-MS because of notable evidence of AML, with four AML-M2 (36%) and three AML-M5 (27%). Four patients (36%) presented as *Class II*-MS due to normal bone marrow, without a prior history of the hematopoietic disorder. No patients with *Class III* and *IV*-MS in our cohort. Patients diagnosed with AML underwent bone marrow flow cytometry. Six (55%) were positive for classic myeloid makers on flow cytometry, including MPO, CD33, CD34, and CD117. CSF flow cytometry in a patient with isolated MS showed positive myeloid markers for CD33, CD117, CD38, and CD64.

**Table 2:** Laboratorial, pathological and genetic characteristics of the patients with MS.

No.	WBC (×10^9^/l)	Hb (g/l)	PLT (×10^9^/l)	PB blast (%)/BM	FCM* positive expression	IHC^#^ positive marker	Chromosome karotype	Molecular testing (Common fusion gene/NGS)
1	7.9	114	311	0/Normal	NA	CD68, MPO, CD33, CD13, CD43	47, XX, add (3) (q21), add (16) (p13) [13]/46, XX [7]	Negative/NA
2	12.3	75	34	76%/AML-M2	CD45, CD34, CD117, CD33, CD13	MPO, CD117, CD33, CD34	45, X, -Y; t (8,21) (q22; q22) [19]/46, XY [1]	*ETO/AML1* positive; *KIT* (*p*.K558 > RGG), *ASXL1* (*p*.S689fs*29), *DHX15* (*p*.R222G)
3	15.4	106	158	48%/AML-M2	HLA-DR, CD33, CD34, CD38, CD117	CD43, MPO, CD117, CD34	46, XY, t (8;21) (q22, q22), add (22) (q13) [20]	*ETO/AML1* positive/Not found
4	9	102	752	0/Normal	NA	Lys, LCA, MPO, CD3, CD34, CD117	46, XX [20]	Negative/NA
5	1.9	78	317	0/AML-M2	CD13, CD15, CD33, CD38, CD117, MPO	LCA, MPO, CD99, CD43	46, XX, del (1) (p34); i (17) (q10) (q13, q13.1) [6]/46, XX	Negative/Not found
6	1.7	112	71	0/AML-M2	CD33, CD13, CD15, CD123, MPO	MPO, CD33	46, XY [20]	Negative/Not found
7	8.0	108	351	38%/AML-M5	CD14, CD64	Lys, LCA, CD43, CD15, CD117	NA	NA
8	18	126	174	51%/AML-M5	CD4, CD11b, CD15, CD33, CD38, CD64	MPO, CD15, CD33	46, XY [20]	*MLL/AF10* positive/Not found
9	16.5	113	286	30%/AML-M5	CD13, CD15, CD33, CD38, CD64, CD117	CD4, CD43, CD117, CD68, CD163, Lys	46, XY [20]	*MLL/ENL* positive/Not found
10	3.2	152	171	0/Normal	NA	CD3, MPO, CD68, CD99	NA	NA
11	4.2	122	179	0/Normal	(CSF) CD33, CD117, CD38, CD64	CD33, CD117	NA	*KMT2A/MLLT3* and *FNDC4/KMT2A* rearrangement; *BCORL1* (*p*.R1332*), *FLT3* (*p*.I836del)

MS, myeloid sarcoma; WBC, white blood cell; Hb, hemoglobin; PLT, platelet; PB, peripheral blood; BM, bone marrow; AML, acute myeloid leukemia; FCM, flow cytometry; IHC, immunohistochemistry; CSF, cerebrospinal fluid; NSG, next-generation sequencing; NA, not available.

* Bone marrow.

# Tumor mass.

### Pathology and IHC findings

**Figure 3:**
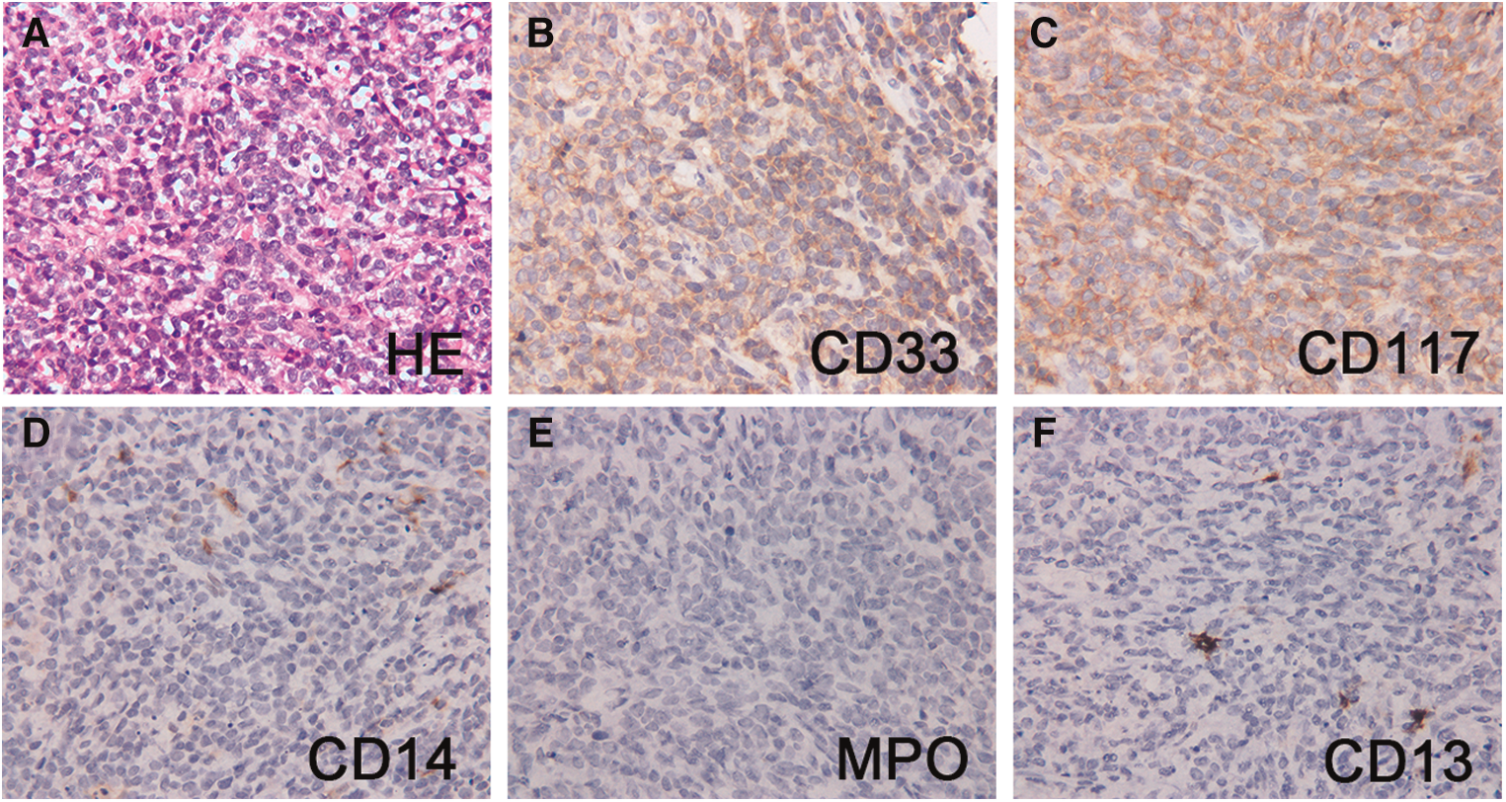
Pathological and immunohistochemical findings of patient 11. H&E stain of a testis mass (**A**) showed mainly small round cells with abundant cytoplasm, large, irregular nuclei, and easily visible nuclear schizophrenic phase. The neoplastic cells strongly expressed CD33 (**B**) and CD117 (**C**), and the blasts were largely negative for CD14 (**D**), MPO (**E**) and CD13 (**F**). Original magnification: 400×.

Pathologic characteristics were presented in Table 2. On hematoxylin and eosin (H & E) staining, MS was characterized by diffuse infiltration of tissues with myeloid cells, a large nucleus, and irregular nuclear contours. Immunohistochemically, MPO (73%) and CD117 (55%) was the most commonly expressed marker, followed by CD33 (45%), CD43 (45%), CD34 (27%), CD68 (27%), and lysozyme (27%). Patient 11 was notable and fell into a diagnostic dilemma because of limited antibody panels and MPO negative. He was not diagnosed with MS until the genetic results of *KMT2A (MLL)* gene rearrangement, a common genetic abnormality of AML, were reported, and an expanded antibody panel retested IHC showed positive for CD33 and CD117 (Figure 3).

### Chromosome karyotype and molecular findings

Karyotyping results were available in eight patients because isolated MS was not routinely examined and one patient with AML refused. Cytogenetic abnormalities were found in four patients (36%) including +3, + 16, + 22, t (8;21), i (17) and del (1). Two patients with t (8; 21) (q22; q22) formed an *AML1/ETO* fusion gene. Despite normal karyotype, two patients were found positive for *MLL/AF10* fusion gene and *MLL/ENL*, respectively. NGS detected mutations in one MS tissue sample, which showed positive for the *KMT2A/MLLT3* and *FNDC4/KMT2A* gene rearrangement, as well as mutations of *BCORL1* (*p*.R1332*), *FLT3* (*p*.I836del). The bone marrow myeloid mutation panel was identified positively in one patient with *KIT* (*p*.K558 > RGG), *ASXL1* (*p*.S689fs*29), and *DHX15* (*p*.R222G).

### Treatment and outcomes

Nine patients received surgical treatment. All patients were treated with chemotherapy based on AML regimens except one who eventually died of disease progression. AML regimens consisting of induction therapy with daunorubicin and cytarabine and consolidation therapy with high-dose cytarabine. CNS-MS patients were managed with central nervous system leukemia (CNSL) regimen. Eight patients (73%) achieved complete remission (CR) after the first or second induction chemotherapy. Of the eight patients, one patient discontinued chemotherapy and relapsed after 4 months w hile one lost to follow-up. Three patients who achieved CR to induction completed hematopoietic stem cell transplantation (HSCT). Of the three patients, two patients achieved sustained remission, and one relapsed 1 year after. One patient was treated with the addition of dasatinib due to the mutation of the *KIT* gene, and another patient with sorafenib for the mutation of the *FLT3* gene. The median follow-up time was 33.5 months in eleven patients. two patients relapsed, one died, and one lost to follow-up. The 2-year OS rate estimated by Kaplan-Meier curves was 90.9% ± 8.7%, and the EFS rate was 64.9% ± 16.7%.

## Discussion

In this study, we investigated a series of pediatric patients with MS at a chinese single institution. We highlights a precise pathological diagnosis based on immunohistochemical staining and the importance of genetic examination as well as early and regular systemic chemotherapy.

According to the single-center data of 11 children diagnosed with MS, the most common subtype was MS accompanied by AML, which supported the previous literature (9–11). Approximately 6.7%–12% of childhood AML patients had MS at diagnosis (12–15). In fact, the incidence is often underestimated because screening for MS in AML patients is not routinely performed in clinical workups. In our cohort, the incidence of MS with AML may be higher because we only included biopsy-proven MS. We also observed 4 patients with isolated MS, a subtype considered to be less frequent in children. MS can mimic many other systemic diseases due to the variety of symptoms caused by the different sites. In this study, the most common sites were the skin, orbital region, and lymph nodes. Patients with orbital MS mostly presented with AML M2 and M5 subtypes, and AML M5 subtype have a higher initial white blood cell count. These are consistent with previous reports (3, 5, 9, 11).

Radiological test plays an important role in the early recognition of MS. CT and MRI imaging can assess the size and location of the tumor and help distinguish it from other lesions such as hematomas or abscesses (16). Consistent with the literature, T1-weighted images were mainly low signal and equal signal, and the lesions were enhanced with different degrees (17). The difference is that the T2-weighted images of patients in our cohort mostly present equal or low signals. Assessment of the efficacy of extramedullary lesions by imaging is also important. However, there is no standard for the time node and frequency of radiological evaluation. Due to the multi-site involvement of MS, some scholars suggested that positron emission tomography/computed tomography (PET/CT) should be the first choice for the imaging evaluation (18–20).

MS, especially isolated MS, is often a challenging diagnosis in pediatric patients (21). Studies have reported misdiagnosis rates as high as 25% to 47% for isolated MS, and inadequate immunohistochemistry is the most common cause (22). In some cases, the diagnosis of MS is not corrected until a bone marrow biopsy confirms myeloid neoplasms. The panel of antibodies for the diagnosis of MS includes MPO, CD14, CD68, lysozyme, CD117, CD11c, CD13, CD33 (23). MPO, CD117, CD13, CD33 reflect myeloid differentiation, while CD68, CD163, CD14, CD11c are expressed in monocytes (4, 24). The addition of CD20, CD79a, CD3 and CD45RO to the antibody panel excludes B-cell and T-cell lymphomas (3, 25). All cases in this study were positive for at least one antigen reflecting myeloid differentiation or monoclear differentiation, with the highest rates of MPO and CD117. MPO is the most commonly used antibody for the diagnosis of MS, with high sensitivity and specificity. However, it is not expressed in MS tumor cells with low differentiation (26). One patient in our cohort was initially diagnosed with another cancer, which also supported the need for a broad antibody group in immunohistochemistry.

Cytogenetic and molecular biological tests play an important role in the ancillary diagnosis, risk stratification and prognostic assessment of MS. Chromosomal abnormalities in 32%–84% of MS subjects (10, 21). The common genetic abnormalities including t (8;21), inv (16)/t (16;16) or t (15;17), which usually suggested a good prognosis (9, 10, 27). *KMT2A (MLL)* gene rearrangements or fusions, chromosome 5 or 7 monosomy, 5q-, and complex karyotypes of chromosomes (three or more chromosome aberrations, except good karyotypes) suggested a poor prognosis and high risk (28–30). Patients with positive *AML1-ETO* fusion gene often presented with orbital masses, while patients with abnormal *KMT2A (MLL)* gene were characterized by leukocytosis, CNS disease, and testicular involvement, which is consistent with the literature (10, 28). NGS of tumor mass may help identify relevant mutated genes in patients with isolated MS to aid in diagnosis, guide prognosis and treatment, like patient 11.

The prognostic data on the involved sites are different in the literature (29). *Johnston* et al*.* showed that pediatric patients with orbital MS and CNS MS presented a lower relapse rate and better outcome than those without extramedullary manifestations (15). Studies suggested that non-skin extramedullary involvement is a favorable prognostic factor for pediatric AML patients (12, 31). In our cohort, one patient with orbital MS had recurrent orbital MS and bone marrow involvement, three patients with skin MS had a good prognosis. Similarly, long-term survival also observed in 2 of 3 skin MS patients in a cohort in Japan (32). Hence, may be more powerful factors, such as molecular characteristics of cancer cells and regular treatments, that determine the prognosis.

Based on previous reports, the treatment of MS is generally systemic chemotherapy, surgery, radiotherapy, HSCT, or a combination of these approaches. Surgery and radiotherapy cannot improve the overall survival of patients but are required when tumors cause obstruction or compression, or leukemia-related skin pain (2, 3, 33). Consistent with the literature (33), patients who received early chemotherapy had significantly longer survival times compared to those who did not receive chemotherapy. Most studies suggested that HSCT after complete remission with chemotherapy improves survival, especially for those who with unfavorable prognostic factors (site, genetic abnormalities, etc.) (2, 3, 9). Two patients in our cohort underwent HSCT after chemotherapy and survived disease free for 22 and 75 months, which also illustrates the importance of HSCT treatment. Targeted agents to improve MS prognosis were also frequently reported such as tyrosine kinase inhibitors (TKI), DNA methyltransferase inhibitors, and CD33 monoclonal antibodies (34, 35). Enhancing the molecular genetic testing of MS patients and implementing individualized treatment may be the future trend.

In summary, MS show a variety of clinical manifestations that can be easily misdiagnosed. Adequate tumor biopsy and immunohistochemistry are necessary for the correct diagnosis of MS. Early and regular systemic chemotherapy promises long-term survival. Due to the rarity of MS, we reviewed too few patients to meaningfully study the impact of potential prognostic factors. It is hoped that future multicenter or even global collaborative large-sample prospective clinical trials will investigate pediatric MS in greater depth.

## Data Availability

The original contributions presented in the study are included in the article/Supplementary Material, further inquiries can be directed to the corresponding author/s.
